# Revisiting the Definition and Recognition of Indigenous Peoples and Local Communities for Biodiversity Conservation

**DOI:** 10.1002/ece3.72958

**Published:** 2026-02-18

**Authors:** Ronju Ahammad, Kamaljit Sangha, Jay Evans, Oscar Metcalfe

**Affiliations:** ^1^ Research Institute for the Environment and Livelihoods, Faculty of Science and Technology Charles Darwin University Darwin Northern Territory Australia; ^2^ Northern Institute, Faculty of Arts and Society Charles Darwin University Darwin Northern Territory Australia; ^3^ Darwin Centre for Bushfire Research Charles Darwin University Darwin Northern Territory Australia

**Keywords:** biocultural, indigenous land management, indigenous land rights, nature conservation, stewardship, traditional ecological knowledge

## Abstract

Globally, there is no single universally agreed‐upon definition of Indigenous Peoples, yet specific criteria are typically used to define whether someone is Indigenous or not, namely self‐identification, historical continuity, linkage to ancestral land and distinctive social, cultural and economic systems. This paper argues that the current definition criteria only act as guiding principles to explain the situation of Indigenous Peoples and does not embrace all Indigenous Peoples. We use three colonial contexts, i.e., countries where colonisers left, settled permanently, and where colonisation did not occur, to explain the current Indigenous Peoples' situation. By drawing the insights from selected cases, we found that either one or two of these criteria, such as cultural and self‐identification, are commonly applied to identify Indigenous Peoples. The cases also showed that recognising rights of Indigenous Peoples to land has been found to offer a positive outcome for conservation and creating socio‐cultural and economic opportunities for the people (e.g., biodiversity conservation, greenhouse gas abatement). We emphasise that not only the definition, but the legal recognition of land rights and involvement of Indigenous Peoples and Local Communities would be of the utmost importance to continue cultural practices attached to their ancestral lands, allowing them to be involved with natural resource management and biodiversity conservation decision‐making, that eventually relates to self‐determination, equity and social and economic justice.

## Graphical abstract

This paper presents how common definitional criteria for Indigenous Peoples – such as self‐identification and cultural distinctiveness – are often used selectively and fall short of recognising their collective land rights. Drawing on case studies, our paper argues that legal recognition of Indigenous land rights is essential for effective biodiversity conservation, continuity of cultural practices and the pursuit of equity, social and economic justice. Recognising Indigenous knowledge systems embedded with cultural and land rights can contribute to conservation science and practices.

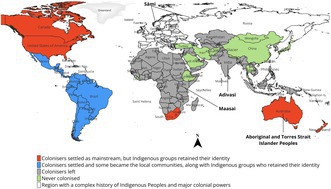

## 1 Introduction

Recognition of Indigenous Peoples and their management of natural systems has gained prominence in recent years, supporting their fundamental rights for survival, dignity and well‐being (Intergovernmental Science‐Policy Platform on Biodiversity and Ecosystem Services [IPBES] 2019, 2022; Rights and Resources Initiative 2023; Stevens et al. 2024). Globally, Indigenous Peoples have experienced systemic marginalisation, stemming from colonisation, forced assimilation and integration, geographic isolation and the imposition of external development models in the evolving socio‐political contexts over centuries (The International Work Group for Indigenous Affairs [IWGIA] 2022; Cambou and Buhmann 2025). Particularly, colonisation has seriously impacted Indigenous Peoples' past hundreds of years by eroding their value systems, cultural knowledge and practices and connections with land, followed now by multi‐national corporations and governments of states where Indigenous groups are in the minority (IWGIA 2024). As a result, the rights to lands that Indigenous Peoples have managed sustainably over millennia, applying their astute knowledge and skills, have been greatly compromised. Given the growing recognition of Indigenous Peoples' role in managing nature and biodiversity conservation, there is a need to appropriately acknowledge their rights worldwide for their own benefit and the benefit of the global community.

In the last half of the 20th century, Indigenous Peoples' rights entered into global discourse, supported by initiatives such as the International Labour Organisation (ILO)'s Indigenous and Tribal Populations Convention, 1957 (No. 107) and an updated ILO 1989 Convention (No. 169), the United Nations Declaration on the Rights of Indigenous Peoples (UNDRIP) 2007 and specialised working groups, including IWGIA and the United Nations Permanent Forum on Indigenous Issues (UN 2009), among several others. The ILO conventions and UNDRIP provide binding and non‐binding frameworks for countries to adopt and protect the rights of Indigenous and tribal peoples universally. However, achieving a single, widely accepted definition of Indigenous Peoples and their rights remains challenging to date due to varying cultural, social and political contexts across different countries, as discussed later in this paper (Peters and Mika 2017). There is no standard definition of Indigenous Peoples. However, a common understanding of who is Indigenous is based on self‐identification and recognition and acceptance by the groups, historical continuity, strong links to territories, distinct language, culture and beliefs, among others (UN 2006).

Today, Indigenous Peoples represent only 476 million, that is, 6.2% of the global population (UN 2025), yet they manage a significant global land mass with low impact. A report by WWF et al. (2021) suggests 32% of the global land and associated inland waters are governed or owned by the Indigenous Peoples and Local Communities (IPLCs) where 80% of those lands are in relatively good condition. A recent report by IUCN suggests that 26% of the terrestrial protected areas overlap with IPLCs' lands (Stevens et al. 2024). Both these reports by IUCN and WWF particularly highlight that those figures are most likely an underestimate since a majority of areas conserved by Indigenous Peoples, or Local Communities, or both in Asia, Africa and Europe are not included yet.

IPLCs' stewardship of their lands and waters has played a major role in maintaining the ecosystems and their services and biodiversity conservation (Dawson et al. 2023, 2021; Russell‐Smith et al. 2009; Sangha 2020; Sangha, Madegowda, and Balasubramanian 2024; Sangha, Ahammad, et al. 2024). Through maintaining these intact landscapes, IPLCs maintain ongoing relationships with nature as a part of their identity, culture and livelihoods and deliver ecosystem services such as regulation of climate and water, prevention from natural hazards, etc. (Sangha 2020). While IPLCs' management of their lands and waters contributes to halting biodiversity loss, they require rights to maintain cultural connections with, and stewardship of nature (UN 2018). Most importantly, appropriate recognition of IPLCs and their rights can enable self‐determination about their political status, own institutions and practices, pursuing conservation, cultural and economic benefits (Donlon 2018; UN 2009, 2021). To date, out of > 100 countries that experienced colonisation in the past only a few countries formally acknowledge Indigenous land rights through constitution or establishing treaties and legislations (e.g., Australia, Brazil, Canada, Congo, New Zealand) that too with limited legal support for implementation (UN 2018).

Throughout history, IPLCs' long‐standing relationship with nature has allowed them to accumulate knowledge on how to govern social‐ecological systems and adapt to changing climatic situations, ecosystems and availability of resources (Berkes 2018). Their Traditional Ecological Knowledge (TEK) is fundamental as it encompasses knowledge, rights, obligations and cultural practices to maintain their physical, cultural and spiritual connection to their ancestral lands, species and protection. Studies reported that conservation scientists can be informed and guided by TEK of IPLCs, which are tied to species and their place‐specific understanding of species distribution (Roué and Molnár 2017; Dorji et al. 2024). While recognition of IPLC's knowledge is on the rise, its use and bridging that knowledge with conservation science and biodiversity planning requires a proper understanding of their land and cultural rights and language, within which their practices exist and contribute to maintaining the stewardship (Connor et al. 2025; Esquible et al. 2025). Recognising and protecting Indigenous cultural knowledge and rights can only benefit future biodiversity conservation if it is protected with the correct set of policies.

This paper seeks to clarify who may be included under the broader definition of Indigenous Peoples in order to enhance conservation outcomes. The way Indigenous Peoples are defined how it impacts legal recognition of peoples' rights, the continuity of peoples' cultural practices and relationships to their ancestral lands, their roles in conservation and natural resource management. This perspective paper analyses key formal definitions of Indigenous Peoples that have been established by global organisations such as the United Nations (UN) and International Labour Organisation (ILO).

In this paper, we will use the term Indigenous Peoples and Local Communities to be inclusive of both recognised Indigenous Peoples as well as Local Communities which in some regions (Asia‐Pacific, Africa) have been inhabiting lands for millennia but are not formally recognised by local governments as Indigenous Peoples [Intergovernmental Science‐Policy Platform on Biodiversity and Ecosystem Services (IPBES 2019)]. We argue that many local communities in the Asia‐Pacific and African regions (especially where colonisers have to leave) have been living in their landscapes for millennia; however, they are often not recognised by their own governments as Indigenous. A good example is India where the colonisers (British) had to leave, and after independence, almost the entire population is Indigenous to their lands in one way or another. However, only those living in the forest are legally considered as Indigenous—tribal people, under the Scheduled Castes and the Scheduled Tribes (Prevention of Atrocities) Act, 1989.

This paper is written in three main parts:

1. The next section describes the definitions of Indigenous Peoples applied by key global organisations and identifies the common defining criteria.
2. Then we analyse how the current definitions apply to the recognition of Indigenous groups in selected national contexts by illustrating examples from Oceania, Europe, Asia and Africa.
3. Finally, we discuss how the definitional criteria and the recognition of Indigenous land rights affect conservation outcomes.

## 2 Definitions of Indigenous Peoples

The earliest formal definition of Indigenous Peoples can be traced to Article 1 of the ILO Convention 169 (1989). This document defines Indigenous Peoples as those ‘whose social, cultural and economic conditions distinguish them from other sections of the national community, and whose status is regulated wholly or partially by their own customs or traditions or by special laws or regulations*’* (ILO 1989). The ILO's definition emphasises the socio‐cultural, linguistic and human rights dimensions of Indigenous identity and underscores the need for binding legal instruments to respect, protect and integrate Indigenous and other tribal or semi‐tribal populations in independent countries. Self‐identification as Indigenous or tribal is the central principle of the ILO definition, ‘self‐identification as Indigenous or tribal shall be regarded as a fundamental criterion for determining the groups to which the provisions of this Convention apply’ (Article 1 of the ILO 1989 Convention). It further emphasises the recognition of Indigenous Peoples' distinctive institutions or socio‐economic conditions, and that their status is regulated wholly or partially by their own customs or traditions. Furthermore, the ILO highlights the importance of recognising Indigenous Peoples' rights to land ownership and calls for government support in identifying and securing their lands.

The United Nations Declaration on the Rights of Indigenous Peoples (UNDRIP) has been an important international instrument that affirms the recognition, promotion and protection of the rights of Indigenous Peoples (UN 2007). The declaration contains 46 articles covering various aspects of Indigenous culture, language and territorial rights, among others, and has been adopted by the majority of states, over 140 countries. However, the document is non‐binding and does not provide any specific criteria to follow for the definition. So, the declaration provides the principles and minimum standards guiding the rights of Indigenous Peoples. For instance, Article 3 recognises the right to self‐determination, and Article 33 recognises the right to determine their identity or membership based on their customs and traditions, aligning with the ILO Convention's focus on self‐identification. UNDRIP further emphasises the protection of Indigenous Peoples' organisations, political and economic institutions, cultures, beliefs, customs and languages. It advocates for harmonious relations between Indigenous Peoples and states, grounded in justice, democracy, respect for human rights, non‐discrimination and good faith, while supporting their self‐governance and active participation.

The UN Special Rapporteur's report, commonly referred to as the ‘Cobo Report’, provides a working definition of Indigenous Peoples (Cobo 1987). The report states: ‘Indigenous communities, peoples and nations are those which, having a historical continuity with pre‐invasion and pre‐colonial societies that developed on their territories, consider themselves distinct from other sectors of the societies now prevailing in those territories, or parts of them. They form at present non‐dominant sectors of society and are determined to preserve, develop and transmit to future generations their ancestral territories, and their ethnic identity, as the basis of their continued existence as peoples, in accordance with their own cultural patterns, social institutions and legal systems’. This definition implies colonial history to assess and compare Indigenous Peoples' continuity of their culture, custom and practices.

Some definitions below are drawn from global Non‐Government Organisations (NGOs), mainly Survival International, International Work Group for Indigenous Affairs (IWGIA), which extensively work with Indigenous Peoples. Survival International defines Indigenous Peoples as ‘descendants of those who lived in a region prior to the dominance of modern societies, with distinct cultures, languages, and worldviews’ (Survival International 2024). This definition explicitly includes tribal peoples, emphasising the group of the population with the distinct identities, dependence on land for livelihoods, self‐sufficiency and limited integration into national societies. As per their definition, tribal peoples are estimated to be about 150 million individuals which constitute 40% of the Indigenous Peoples globally, sharing a profound attachment to the lands for their livelihoods. IWGIA proposes the term ‘Indigenous Peoples’ as a collective identifier for distinct groups that have been historically marginalised and denied the right to control their development (IWGIA 2018). IWGIA emphasises collective rights to land and natural resources, with self‐identification recognised as a fundamental criterion for claiming Indigenous identity.

More recently, the Intergovernmental Science‐Policy Platform on Biodiversity and Ecosystem Services (IPBES) applied the term Indigenous Peoples and Local Communities (IPLCs) (IPBES 2019). They mentioned IPLCs as ‘typically ethnic groups who are descended from and identify with the original inhabitants of a given region, in contrast to groups that have settled, occupied or colonised the area more recently’. IPLCs maintain an inter‐generational historical connection to place and nature through livelihoods, cultural identity, languages, worldviews, institutions and ecological knowledge. Indigenous Peoples are regarded as First Peoples, Aboriginal Peoples, Native Peoples, or Autochthonous Peoples, descended from and identifying with the original inhabitants of a given region, and a local community can comprise heterogeneous ethnic groups with their relation to and collective knowledge of place, territory and the environment they live in. IPBES also suggested the local communities as ‘non‐Indigenous communities who are highly diverse and recognised for having historical linkages to places and natural resources, multiple domains of ecological knowledge, dynamic and hybrid resource management techniques and technologies, customary and formal institutions to manage natural resources, and distinctive worldviews and relationships to nature’ (IPBES 2022).

Among various definitions, the ILO and IWGIA emphasise land rights significantly, whereas UNDRIP prioritises self‐identification and cultural preservation. While the ILO provides a legally binding framework for the protection of Indigenous rights, UNDRIP offers non‐binding principles that serve as a guideline for states. Survival International uniquely includes tribal peoples within its definition, expanding the scope to recognise related but distinct groups. The ILO and the UNDRIP focus specifically on Indigenous Peoples, while IPBES extends the concept to include Local Communities. IPLC conception used by IPBES primarily focuses on the commonalities and shared concerns of Indigenous Peoples and Local communities in connection with land and biodiversity, however we also acknowledge the concern of overgeneralising the term may conflate with the distinct rights of Indigenous Peoples (Huambachano et al. 2025). Most notably, the Cobo (1987) (the UN Special Rapporteur's report) highlights the importance of ‘historical continuity’ criterion which is not mentioned in any other definition. Historical continuity with pre‐colonial and pre‐settler societies and Indigenous Peoples' strong link to territories and surrounding natural resources apply to identify them as ‘Indigenous’.

Drawing on those definitions, we selected five key criteria that emerged as more or less common to define Indigenous Peoples. Those include Self‐identification; Historical continuity with pre‐colonial and pre‐settler societies and strong link to territories and surrounding natural resources; Distinct language, culture and beliefs; Distinct social, economic or political systems; and Minority/Non‐dominant groups of society (Table 1).

**TABLE 1:** Evaluation of Indigenous Peoples and Local Communities' definitions using a set of five key criteria from each of the selected global definitions and the inclusion of local communities (see details for definitions in Data S1).

Definition sources	Self‐identification	Historical continuity	Distinctive social, economic and political systems	Distinctive culture and language	Minority/Non‐dominant status
ILO (1989)	√	X	√	√	X
UN (2007)	√	X	X	X	X
Cobo (1987)	√	√	√	√	√
Survival International (2024)	X	X	√	√	X
IWGIA (2018)	√	X	√	X	√
IPBES (2019)	√	X	√	√	√

Self‐identification and distinctive social, political and economic systems, culture and language are commonly mentioned criteria to define Indigenous. Cultural criteria encompass specific language, religion, tribal system, membership of an Indigenous community, residence in certain parts of a country or certain regions are formally mentioned to identify Indigenous Peoples globally. While cultural criteria are deeply entrenched with all other elements, the ancestral land and connection have fundamental importance for their physical and cultural identity, customary laws and sustainable use of natural resources.

## 3 Implications of the Current Indigenous Peoples' Definitions

Currently, there are no clear guidelines and criteria for who is considered Indigenous, especially when estimating the 476.6 million Indigenous and tribal peoples globally based on self‐identification (ILO 2019). Self‐identification of Indigeneity, as advocated by the UN, including IPBES^1^, UNEP and other global organisations, is a widely applicable criterion, but can be quite confusing, and impede communication and policy development.

We explain Indigeneity in three colonial‐geographical contexts. Indigenous Peoples of:

1. Countries that were colonised and then colonisers had to leave (such as India, Pakistan, Indonesia).
2. Countries which were colonised and then colonisers became settlers (such as Australia, Canada, United States, New Zealand, Brazil and Chile).
3. Countries that were never colonised by external parties (only a few—Afghanistan, Bhutan, China, Ethiopia, Iran, Japan, Liberia, Mongolia, Nepal, Saudi Arabia, Tonga, Turkey, Thailand).

Here, we apply the Indigenous Peoples’ definitions to the first two contexts to explain how they create confusion. In the first instance, where colonisers had to leave, we argue that all local people who are citizens of that country are native/Indigenous as they belong to ‘a’ place in that country they have lived in for generations. Some of them, such as urban populations, may have lost traditional practices and traditions, in particular their connections to land; however, a majority of them, especially those in rural and remote areas, have kept their traditions and connections with their lands. In such instances, where the population of a country belongs to that geographical place, it is ironic for them to demonstrate/declare their Indigeneity. It may be worthwhile to understand the populations that have maintained cultural traditions, language and connections with land.

In the second instance, where colonisers became settlers along with the local/Indigenous population, it is relatively easier to apply the definitions mentioned earlier due to a clear distinction between the Indigenous population of a country and those who settled there later. A good example is Australia, where there is a clear distinction between the local‐Indigenous and non‐Indigenous populations. Within the settler population, then the question comes if they have become one of the local communities for living there over a longer period of time such as people from Spanish background who settled in south America a few centuries back.

**FIGURE 1:**
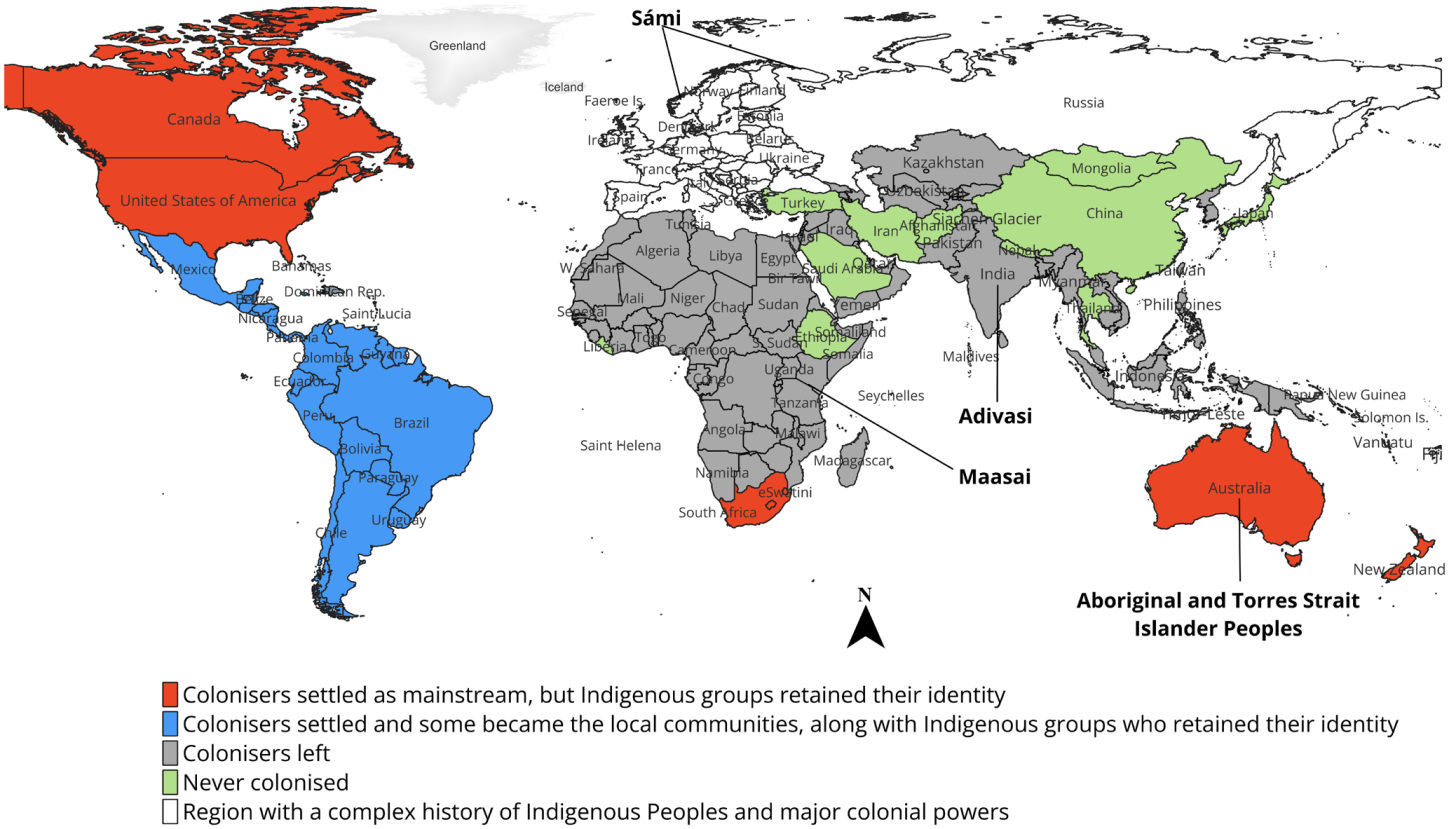
Illustrating three colonial–geographical contexts that lead to different outcomes for recognising/self‐identifying Indigenous Peoples across the globe for 1900s to date. The Blue area covers Southern and Central America where Indigenous Peoples and Local Communities are somewhat differentiated. Indigenous Peoples are the original people from those areas while Local Communities may include settlers who have been living there for some ×100s of years. The Red area represents Australia, New Zealand, United States of America and Canada and some other countries—where a clear distinction between Indigenous Peoples and non‐Indigenous peoples exists, hence the current definition mainly applies. In Grey areas of the map covered Asia and Africa—most of the population is native/Indigenous who have been living there for millennia, while only a tiny proportion is recognised as Indigenous Peoples, if any. The White area accounts for the key colonial powers and is also inhabited by Indigenous groups across several countries without defined boundaries. In this figure, our purpose is mainly to highlight the difference between countries where colonisers are currently part of the place, and where they have to leave the country (ruled for administrative purposes).

In the third instance, there is no issue of colonisers as the entire population of a country will be Indigenous. This is true for countries such as Afghanistan, Bhutan, China, Ethiopia, Iran, Japan, Liberia, Mongolia, Tonga, Turkey, Nepal, Thailand. We illustrate this using the map above, which indicates different forms of colonisation that shape IPLCs' context (Figure 1). The map is developed to provide a general overview of the Indigenous Peoples and Local Communities under different colonisation contexts.

We demonstrate the first case by using the example from the Asia Pacific region (Figure 1) where a large majority of the population (> 90%) is Indigenous in most of the countries in the region. However, their recognition at the government/political levels is either lacking or limited to only those living in forest areas. India is a good example where roughly > 99% of the population is ‘originally’ from that land, commonly called ‘Hindustan’/‘Bharat’ (in the ancient names) and this point is also mentioned in India's reply to UNDRIP (Bijoy et al. 2010). However, currently, only a small 8%–10% of the total > 1.4 billion population is recognised as Indigenous under the title of ‘Adivasi*’*, ‘scheduled tribes’ or ‘tribal’ peoples [under the Scheduled Castes and the Scheduled Tribes (Prevention of Atrocities) Act 1989]. The question is what about the rest of the population who is also native to the same land. Particularly, rural communities such as farmers and farm labourers, who still hold strong traditions, language, customs and cultural values through ongoing connections with their land and water systems. The current Indigenous Peoples’ definitions fail to embrace them.

Hence, the number 476 million Indigenous Peoples (as mentioned earlier) may indeed represent a gross underestimate of what is the real number of Indigenous Peoples across the globe—implying some serious consequences for both Indigenous Peoples and Local Communities (IPLCs) which subsequently impact the use and management of natural resources and related conservation outcomes.

## 4 Case Studies of Applying Current Indigenous Peoples' Definitions

We selected four cases from Australia, Scandinavia, Russia, Africa and India, where colonialism and post‐colonial states shape the historical, socio‐political and cultural context of Indigenous Peoples, and thus their recognition to varying degrees. The geographical location of Indigenous Peoples of the four cases covers their spread across country (Adivasi in India) to regional (Sámi population in Nordic Countries and Russia), border region (Maasai people in Kenya and Tanzania), and the Aboriginal people in the continent of Australia. The historical context of Aboriginal Peoples in Australia and the Sámi population in Nordic Countries and Russia is relatively different from that of Adivasi in India and the Maasai people of Kenya and Tanzania. The following section illustrates the application of the definition criteria in Table 1 within the context of Indigenous Peoples.

### 4.1 Aboriginal or Torres Strait Islander Peoples in Australia

Australia follows three parts of the criteria (Table 1) including descent, self‐identification and community acceptance to define Indigenous Peoples, often called Aboriginal and/or Torres Strait Islander people. The Aboriginal and/or Torres Strait Islander people account for 3.8% (983,700 people) of the total Australian population (Australian Bureau of Statistics 2021). In the 1980s a definition was proposed in the Constitutional Section of the Department of Aboriginal Affairs’ Report on a review of the administration of the working definition of Aboriginal and Torres Strait Islanders (Commonwealth of Australia 1981, cited in Department of the Parliament Library 2000) as: ‘An Aboriginal or Torres Strait Islander is a person of Aboriginal or Torres Strait Islander descent who identifies as an Aboriginal or Torres Strait Islander and is accepted as such by the community in which he (she) lives*’*. Another important legislation Section 3(1)(g) of the Aboriginal Land Rights (Northern Territory) Act 1976 (Cth) defines an Aboriginal as ‘*a person who is a member of the Aboriginal race of Australia* (descent)’ (Department of the Parliament Library 2000). A distinctive aspect in Australia is the legal flexibility, especially for individuals with disrupted genealogical records (e.g., members of the Stolen Generations), in the face of disruption, to identify them as the race of Indigenous Peoples (Sutton 2009). Although Australia does not apply the historical continuity criterion, the legislation allows individuals/groups to claim land rights and maintain their ancestral linkage. The important legislation regarding Indigenous rights to land includes the Aboriginal Land Rights (Northern Territory) Act 1976 (Cth) and the Native Title Act 1993 (Cth).

Initially Australia did not support the UNDRIP which first came out in 2007 after 20 years of negotiation between states and Indigenous Peoples' representatives around the world. Later, in 2009, Australia endorsed UNDRIP although the rights set out in UNDRIP have not been comprehensively adopted in national law and practice (Delaney et al. 2020). Notably, Australia has not ratified the ILO Convention 169, which provides a legally binding obligation for the state to protect the rights of Indigenous Peoples through integrating the conventions into law and practices and its provisions complement UNDRIP (Commonwealth of Australia 2023). However, during colonisation over the last century, the traditional way of life, cultural and spiritual values, and traditional governance of Indigenous Peoples have endured agony, disruptions and discrimination. During the process of colonisers' settlement and that distress, adversity and affliction still exists in various forms (Sutton 2009).

### 4.2 Sámi Indigenous People in Sweden, Norway, Finland and Russia

The Sámi are the Indigenous Peoples of the northern part of Sweden, Norway, Finland and large parts of the Kola Peninsula in Russia. Following the global definitions, only relevant criteria for the Indigenous status of the Sámi people are tied to their language and cultural aspects (Berg‐Nordlie 2022). Each State has passed laws covering linguistic and cultural and other rights protection (e.g., Sámi Language Act, Norway's Finnmark Act 2005 and the Federal Law on Guarantees of the Rights of Indigenous Small‐Numbered Peoples of the Russian Federation). The constitutions in these four states ensure certain protections of the rights for cultural and language development, with Finland and Norway's constitutions only recognising the status of Sámi as Indigenous, while the Russian constitution recognises the 47 ‘Indigenous Small‐numbered Peoples’ (Swift 2025). Despite the mention in the constitution recognising Sámi as Indigenous Peoples for the protection of their cultural practices, there is yet no legal protection of the Sámi culture on the ground (Mörkenstam 2019). We acknowledge the discrepancies between the promises of national legislation and the realisation of the rights of the Sámi people in practice (Cambou and Ravna 2024a). Sweden, Norway and Finland endorsed UNDRIP, while Russia abstained; and among the four states, only Norway ratified ILO Convention 169 to recognise Sámi as its Indigenous Peoples (Cambou and Ravna 2024b).

In Sweden, Norway and Finland, individuals must declare themselves as Sámi and meet language or lineage requirements. The criterion for individuals to identify as Sámi is to either speak Sámi languages at home or have at least one Sámi parent or grandparent who speaks Sámi. Other local cultural criteria exist to identify this group through their reindeer herding rights. The right is restricted to Sámi individuals who descend from families traditionally involved in herding, showing a blend of descent and economic‐cultural practices. However, there is an absence of ethnic data about Sámi who identify with the Sámi heritage in the census in Norway, Sweden and Finland; most countries rely on the voter lists from Sámi parliaments and research‐based cohorts (Dawson 2020). This process obscures the identity of Sámi people and any reliable estimate and updated information about Sámi heritage. The Sámi people are estimated to be between 50,000 and 100,000 in 2021 across these four countries (50–60,000 in Norway, 20–40,000 in Sweden, 8000 in Finland and 2000 in Russia) (IWGIA 2023).

The Sámi Parliament Acts resulted in the creation of Sámi parliaments, which were intended to ensure self‐determination through political and social development in Nordic countries (Kuokkanen 2024). However, the parliament is not independent of state oversight, nor does it possess decision‐making authority over policies affecting the Sámi people (Allard 2018; Raitio et al. 2020). The Sámi parliaments of Finland, Norway and Sweden drafted the Nordic Sámi Convention to strengthen Indigenous rights, but as of May 2025 it remains unratified and the parliaments hold only advisory, not legislative powers. Sámi parliaments are represented by the groups to raise their rights and interests in the use of lands and water. However, the participation of the Sámi people and their self‐determination has not been part of the Act or country's legislation (Kuokkanen 2024). By contrast, the Sámi population has no parliamentary representation in Russia, where they are classified as Indigenous Peoples with other ‘numerically small’ minority peoples. Indigenous Small‐numbered Peoples, including Sámi in Russia, are defined as those groups with a combined total of less than 50,000 people, living in their traditional ancestral territories, adhering to a traditional way of life and identifying as a distinct ethnic group (IWGIA 2023). Due to the restrictive population size which includes a numerically small number of people in the North, Siberia and the Far East, Russia's definition excludes other Indigenous groups whose number goes above 50,000, like the Yakuts (~400,000 people).

### 4.3 Scheduled Tribes (Adivasi People) in India

Indigenous Peoples in India are covered under ‘Scheduled Tribes’ or Adivasi (original inhabitants of the land) (Dhir 2015), comprising 705 ethnic groups from 30 states in the country. Adivasi people comprise around 8.6% of the total Indian population (IWGIA 2024). Most of the Adivasi belong to the scheduled tribes, some to the scheduled castes and others with lower possession of education and control of the social, economic and political system (Gupta 2024). However, some ethnic groups are not officially recognised within this Scheduled Tribal status, particularly six vulnerable tribal groups^1^ who are deprived of government provision for access to jobs, education and the protection of atrocities through receiving justice (IWGIA 2025). However, the vulnerable groups were not recognised as the government's mega development project expanded into the biodiversity‐rich areas inhabited by the tribes. The most common criteria applied to recognise these tribes, in line with global definitions, include historical continuity, cultural distinctiveness in terms of language, religious and social practices and economic and political marginalisation.

India has ratified UNDRIP in 2007, and the ILO Convention No. 107 on Indigenous and Tribal Populations (1957), but not ratified the more progressive ILO Convention No. 169 (Dhir 2015). While India voted for the UNDRIP in 2007, the government continues to deny the term and concept of ‘Indigenous Peoples’ claiming that all Indians are Indigenous (Bijoy et al. 2010). The Indian government applies several constitutional provisions to protect the tribes and their self‐governance. Especially under Article 342 of the constitution, the President of India notifies and defines tribes or tribal communities as Scheduled Tribes. The 5th and 6th Schedules of the Constitution offer special laws on Indigenous Peoples' land rights and self‐governance. The Constitution also acknowledges Fundamental Rights and prohibition laws that combat discrimination and promote equality before the law, such as non‐discrimination against any citizen on grounds of religion, race, caste, sex, place of birth, while offering special provisions for people from Scheduled Castes and Scheduled Tribes (UN 2008). However, to address equity and other socio‐economic disadvantages faced by Scheduled Tribes, the Indian Constitution includes privileged access to legislation, government jobs and educational institutions. The Scheduled Tribal communities have also been allowed a greater degree of autonomy in their territories, particularly in central and northeastern India (under the 5th Schedule of the Constitution).

### 4.4 Maasai People of Tanzania and Kenya

Indigenous Peoples comprise several multicultural ethnic groups in Africa, including hunter‐gatherers and nomadic and semi‐nomadic pastoralists (ACHPR and IWGIA 2005). Our focus is on Maasai people who are semi‐nomadic pastoralists inhabiting the border through southern Kenya and northern Tanzania. The Maasai population is estimated to be around 1,189,522 in Kenya and 430,000 in Tanzania (as of 2018), although there is no reliable census information (Downie 2022; Minority Rights Group International 2018). Maasai people are known for their distinct dress, culture and traditional cattle‐herding livelihoods.

The global definition criteria, such as the distinctive socio‐economic system, non‐dominant, distinct culture and language, apply to the Maasai people as Indigenous. However, such criteria are not practical to apply at the state level as both dominant and non‐dominant/marginalised ethnic groups are considered Indigenous to Africa (Barume 2010). Tanzania has 125–130 ethnic groups (tribes) in the country, and the Maasai people are one of them (IWGIA 2024). One of the contentions is that the Maasai people are not called ‘original inhabitants’ of the area, in contrast to the case of Pygmies, San and the Hadzabe communities. The Maasai were relatively recent pre‐colonial arrivals to the region but nonetheless maintain close connections to the land. In this case, the global definition criterion on the historical continuity of the pre‐colonial inhabitants of the land contradicts African contexts as it is not unique to just one group in Africa. Most African peoples can demonstrate historical continuity, although some groups were highly mobile than others in the past (Hitchcock 1993). Maasai people occupied areas of Tanzania for generations, building cultural ties to the land prior to European colonisation, and thus self‐identify as Indigenous.

Rather than calling one group Indigenous to Africa, the African states have adopted the word ‘Tribal’ to describe diverse ethnic and cultural groups. The African Commission on Human and Peoples' Rights has applied the term in a way that includes various aspects of Indigenous Peoples aligned with the international definition (Tamuno 2017). Indigenous and Tribal peoples are interchangeably used to avoid confusion, and more emphasis is given to peoples' cultural attachment to land during the pre‐ and post‐colonisation periods. The African Commission specifically emphasised dependence on land for survival, marginalisation in terms of dispossession from land and self‐identification as the distinct criteria for African indigenousness. The Commission insisted that the Indigenous concept is needed in Africa to address the situation of marginalised peoples.

Kenya has neither ratified the UNDRIP nor ILO Convention 169, although its constitution contains a progressive Bill of Rights regarding the protection of minorities and marginalised groups (IFAD and IWGIA 2022; IWGIA 2023). On the other hand, Tanzania has only voted for UNDRIP but did not ratify the ILO Convention. In both countries, Maasai people face challenges in land rights and cultural practices due to the lack of specific legal provisions.

## 5 The Way Indigenous Peoples are Defined Impacts Indigenous Land Rights and Biodiversity Conservation

The definitions, especially UNDRIP and ILO Convention 169, emphasised the recognition and protection of the rights of lands and water pertaining to Indigenous Peoples' cultural and customary practices to use the resources. Historical continuity and cultural distinctiveness help establish clear connections to the land and traditions that underpin Indigenous Peoples' rights and claims on natural resources. These criteria reinforce the legitimacy of their role in issues like conservation. Here, we briefly discuss the recognition of land rights in all four selected cases, particularly how Indigenous Peoples sustainably manage ecosystems.

First, in the case of Australia, the recognition of Indigenous land rights tends to improve conservation outcomes (Farr et al. 2016). The legal recognition of native land titles under Indigenous land and sea frameworks has resulted in Indigenous communities managing over half of the protected areas in Australia (1 million km^2^ as of 2022). Notably, Indigenous areas support the protection of habitat for half of the threatened species (out of the total of 272 terrestrial or fresh water vertebrate species) in the country (Renwick et al. 2017). Most importantly, Indigenous protected areas management has been seen as a critical step to the cultural and economic self‐determination of Aboriginal and Torres Strait Islander people (Country Needs People 2016; Farr et al. 2016). Indigenous Peoples develop the plan addressing local and national priorities of biodiversity conservation and their cultural management practices embrace the connections to land and sea and offer opportunities for improving health, education, employment and cultural identity.

In the second case, Sámi Indigenous People maintain their cultural connection to the homeland (Sápmi) for reindeer herding, hunting and fishing – especially the traditional foraging routes for herds in Sweden, Finland and Norway (Allard 2023). Reindeer herding is a traditional, collective, nomadic livelihood and cultural practice of the Sámi people that exists on suitable pasture areas in different seasons (Finland is an exception, where reindeer herding is also permitted for non‐Sámi people). In Sweden, Sámi reindeer herding areas cover over half of the country's land surface (Sandström 2015). Although they are generally given the land usage rights for reindeer herding/husbandry, hunting or fishing, there are no designated reserves or areas and permanent land title (Raitio et al. 2020). Without permanent land rights, Sámi people have mostly been denied self‐determination and cultural autonomy in decision‐making of the conservation and natural resources on their traditional territory. The particular concern has been a lack of specific state legislation to maintain the international standard of Free, Prior and Informed Consent (FPIC) of Indigenous Peoples about any granting of extractive resource practices on their land or territories. In particular, in the Swedish context, there are no specific rules or legislation requiring consultation with Sámi about the impacts of land use (Allard 2018). Multiple land use pressures, primarily intensive forestry, industrialisation (facilities for wind power, mining) and infrastructure development have fragmented the traditional landscape with less available and accessible reindeer‐grazing areas on Sámi homeland (Allard 2023; Stoessel et al. 2022).

In the third case from India, tribal people and forest dwellers (i.e., Adivasi) contribute to protect many forest areas including sacred groves across the country due to their religious and cultural connections, and also contribute to biodiversity hotspots. Adivasi districts account for over half of India's forestlands (~60%) whereas ~38% of the land cover in each district is forested (Gupta 2024). Historical continuity in these practices prevents deforestation and maintains habitats. The Forest Rights Act (2006) (also known as the Scheduled Tribes and Other Traditional Forest Dwellers (Recognition of Forest Rights) Act (2006)) is the first of its kind for recognising Adivasi peoples' forest rights through access to land, minor forest products, water bodies and other resources. However, this act only allows for the use of resources and not the right to determine and regulate the forest land use from external interests. Despite the recognition of the rights of Adivasi, the diversion of forest lands to non‐forestry purposes and management authorities from local communities continues.

Fourth, the Maasai people's traditional pastoralism relies on rotational grazing systems to balance pasture use and regeneration, thereby sustaining the African savanna ecosystems. Maasai people have not received any legal recognition of land rights from Kenya and Tanzania governments despite their role in preventing land degradation and biodiversity conservation (Barume 2010). The establishment of national parks led to the disposing of their land rights for herding and maintaining cultural practices, including visits to spiritual sites (Melubo 2020). The government of Tanzania enacted land laws to improve land access and ownership, which generally promoted privatisation of land, without making any efforts to protect the collective rights of the Maasai to use pastoral commons (Seno and Shaw 2002; Sundstrom et al. 2012). In recent decades, the Maasai have faced challenges related to land rights, as their grazing lands are increasingly being converted for agriculture or tourism, affecting their traditional knowledge, use of pastoral lands and wildlife conservation (Kegamba, Sangha, Wurm, Kideghesho, and Garnett 2024; Nkedianye et al. 2020). Disruptions to traditional pastoralism practices and rights, often resulting from land privatisation, have led to increased dispossession, land degradation and biodiversity loss (Weldemichel 2022). The relatively recent pre‐colonial arrival of the Maasai to the region (and displacement of other ethnic groups) highlights the question of historical continuity in defining Indigenous Peoples.

### 5.1 Recognition of Indigenous Peoples and Local Communities' Knowledge Systems to Inform Conservation Science and Practice

In Australia, savanna fire management draws on many thousands of years of Indigenous Peoples' cultural practices and knowledge. Indigenous practice creates a patchy burnt landscape and natural firebreaks that limit the extent of destructive wildfires in the hot late‐dry season. Indigenous fire management was discontinued with people's displacement from their ancestral lands during colonisation. Since the 1970s, with the progress to self‐determination of Indigenous Peoples and gradual reoccupation of homelands, Indigenous Peoples have reinstated cultural burning as a part of land management to protect the environment and reduce uncontrolled wildfires that posed serious threats to biodiversity (Russell‐Smith et al. 2009). Remarkably, since the 1990s, the importance of cultural burning has grown with a collaborative experiment between Indigenous ranger groups, Indigenous landowners and Western science that created an innovative savanna burning methodology to account for the reduction or avoidance of greenhouse gas emissions (lesser fuel load and extent of wildfire) and biodiversity loss. While the practice contributes to the Australian government's GHGs emission commitment, it was only possible with Indigenous Peoples' access to land management, Native land titles and community‐led aspirations and cultural obligations. Mostly, the growing recognition of traditional practice allows Indigenous Peoples to access economic opportunities, build key partnerships (including with their local Indigenous ranger programs), build capacity and continue sharing cultural knowledge with younger generations (Ansell and Evans 2019).

Traditional knowledge and practices contribute to the protection and regeneration of forests as 60% of India's forests are in the Adivasi (Scheduled tribes) districts (Gupta 2024). For instance, the local tribal population ‘Soliga’ maintain knowledge about traditional land use practice like mosaic burning, followed by fallow agriculture, sustainable forest harvesting for subsistence use guided by their cultural norms and rules that they do not degrade sacred sites and habitat for megafauna in the Western Ghat biodiversity hotspot (Sangha, Madegowda, and Balasubramanian 2024). The Soliga hold fine scale knowledge of movement and foraging habits of tigers ( *Panthera tigris* ) and Asian elephants ( *Elephas maximus* ) in the landscape. Traditional ecological knowledge of the Soliga enables them to locate places for collecting honey from bees or identify specific trees in the forest, minimising people's confrontation, hence promoting coexistence with wild animals. However, India's existing Forest Conservation Act (1980) undermines any such traditional knowledge, cultural rights and community‐based decision‐making about forest use and management by placing the power in the hands of the government rather than tribal and local communities (i.e., village assembly). While the Forest Rights Act (2006) recognises the ‘right to protect, regenerate, or conserve or manage any community forest resource, it does not guarantee the traditional practices’. Like Soliga and other Adivasi people in India, the recognition of their Indigenous knowledge and cultural practices can achieve conservation outcomes if they are allowed the rights to apply their knowledge in managing the forests in which they live, with support from government and non‐government authorities.

Sámi reindeer herders' worldviews and traditional knowledge about maintaining the relationship between human, reindeer and nature are unacknowledged within the Nordic states' policies (Johnsen et al. 2017). The traditional management of Sámi reindeer pastoralism is that a high level of herd diversity (i.e., age, sex, colour, body type and temperament of the animals) reduces vulnerability and enhance adaptation of the whole herd in unfavourable grazing conditions and environment (Berkes 2018). In the past several decades, the Nordic governments promoted reindeer husbandry with the modern high‐yield (pure, more females) variety of reindeer with economic incentives and sanctions that undervalued the traditional herding practices through observation, mobility and maintenance of heterogeneity in herds for ‘buffers’ (Degteva et al. 2023). Despite Norwegian government ratified the ILO Convention 169, the state's scientific approach and policy through new reindeer breeding undermines Sámi people's rights to control their culture and traditional knowledge of reindeer herding. Similarly, in Finland, the non‐exclusive rights of reindeer herding to Sámi people do not recognise the traditional connection of Sámi with reindeer, the land and the knowledge for sustainable pastoral land use (Ott 2025). While the government of Sweden recognised the Sámi people as an Indigenous people in 1977 and acknowledged reindeer herding as a central part of culture, there is no recognition and support within the forestry legislation for the Sámi in making decisions on homeland (Brännström and Ravna 2024). Despite 40 years of recognition of the rights to a Sámi way of life, it is not clear how the rights could or should be translated into practical land management and biodiversity conservation.

The Traditional Ecological Knowledge of the Maasai people encompasses the communal practice of collecting medicinal herbs from their pastoral commons, in situ learning and the application of these herbs with the advice of elders and knowledgeable pastoralists (Hedges et al. 2020). Throughout the 19th century (pre‐and post‐colonial formation of state government), the formalisation of communal land tenure (shift from communal pastoral lands to private land tenure) resulted in changing the locality of living and reduced access to plants both physically and temporally (Kieyah 2007). With the land tenure enforced on the Maasai people through privatisation, the division of their customary pastoral land has under addressed the source of medicinal plants in the landscape and the traditional knowledge, language and cultural practices associated with their use. The breakdown of distinct cultural practices rooted in customary management of pastoral commons, combined with the loss of intergenerational knowledge about the plants, resulted in a nearly 40% decline in the active use of medicinal plants and their conservation (Bussmann et al. 2018).

## 6 Discussion and Conclusion

By drawing the insights from the various definitions of Indigenous Peoples, it is evident that no universal set of criteria applies to define Indigenous Peoples or Local Communities. Diverse historical, cultural and political contexts affect policy and legislation in recognising the land rights and the outcomes for Indigenous and non‐Indigenous communities/peoples globally. The definitions and their constituent criteria provide a global understanding of the key identifying characteristics of Indigenous Peoples regardless of how any government follows and justifies its position with policy, legislation, etc., in addressing the cultural, political and economic context of Indigenous peoples within the state. As a result, governments only ratify the global conventions, for example, UNDRIP or ILO Convention 169; however, that does not guarantee protection of Indigenous rights to lands or their self‐determination on the use or management of natural resources. In all the countries reviewed here, legislations are made to protect lands primarily for conservation, without a focus on safeguarding the language, cultural practices and knowledge of Indigenous Peoples in connection to the lands.

While the policies and Acts recognising Indigenous land rights are on the rise to some extent, their implementation is not aligned with the obligations of the ILO Convention (Ravna 2016). Even when legislation exists for legal recognition, it does not conform to the customs and Indigenous tenure systems to protect their right to use and manage. Indigenous Peoples are not given full authority for decision‐making and thus do not have substantial control over the natural resources. Looking at the Nordic case, neither of the existing Acts (e.g., Finnmark Act, 2005, the Reindeer Herding Act, 2007 in Norway or similar Acts in Sweden and Finland) allows the Sámi population to have the right to make management decisions of their traditional grazing lands (Josefsen and Saglie 2024; Kuokkanen 2024; Ravna 2015). Having no legislative protection of the traditional land rights, the growing extractive industries and the establishment of wind power plants have affected the availability of pasture lands, cultural rights and the livelihoods of Indigenous Peoples (Allard 2023). Similarly, the Scheduled Tribal populations in India do not have any rights or ownership of the lands to make decisions about the natural resources under the Forest Rights Act (2006) (Loivaranta 2023).

The absence of formal recognition of Indigenous Peoples and their land rights or the presence of overlapping land claims undermines Indigenous participation, intensifying pressures from external land investments (i.e., mining, renewable energy site), resulting in ecological degradation and loss of traditional cultural practices, knowledge systems and livelihoods (Larsen et al. 2022). No clear rules are available for compensating and sharing royalties from mining on Indigenous land and the loss of cultural, health and well‐being of Sámi people (Larsen et al. 2022). The exclusion of tribal peoples' land rights has several ramifications for the internal displacement of tribal groups from their ancestral, culturally important lands that they maintain for biodiversity and their subsistence livelihoods (Chakma 2024). In Africa, the Maasai people miss out on rights to receive financial benefits from tourism activities from the most famous national parks and wildlife conservation areas. Maasai existence is further threatened by land grabbing due to a lack of secured and enforceable land rights on their traditional lands in Tanzania and Kenya (Kegamba, Sangha, Wurm, Kideghesho, and Garnett 2024; Kegamba, Sangha, Wurm, Meitamei, et al. 2024; Timmins et al. 2022). By contrast, Australia shows a growing formal recognition that the legal provision of land tenure‐related rights can enhance Indigenous Peoples' cultural, political and economic rights on land management (Farr et al. 2016).

The importance of recognition of cultural expressions of life, language and ecological knowledge attached to land facilitates understanding and identifying Indigenous Peoples. Nonetheless, cultural recognition in itself does not ensure Indigenous Peoples' self‐determination about the use of their lands. The Free, Prior and Informed Consent of Indigenous Peoples can underpin their political and economic participation in all stages of conservation and development activities on their lands (Kennedy et al. 2023). Sámi Indigenous status allows their minority rights and cultural autonomy related to language and education, rather than the recognition of land rights. Despite the progress in cultural rights recognition, many tribal groups in India could not avoid the dispossession of their traditional lands from industrial development, mining and deforestation, which neither benefits their livelihoods nor the environment (Bose 2023). Native title recognition in Australia, especially with exclusive rights and land rights legislation allow Indigenous Peoples' connection to and authority over their land (Hill et al. 2013). Overall, the selected four cases indicate that secure tenure with practical governance and management of land by the Indigenous Peoples is critically important for strengthening their short‐to long‐term capability and socio‐economic well‐being. Both cultural autonomy and self‐determination for Indigenous Peoples can contribute to inclusive land use and community‐based conservation decision‐making processes on Indigenous lands.

Worldwide, Indigenous Peoples' efforts to manage and protect nature and its resources over a quarter of global land area are well recognised (Garnett et al. 2018). While Indigenous Peoples have received internationally recognised rights through UNDRIP and ILO, the number of Indigenous Peoples, if appropriately recognised, can be much greater and can cover much greater area under conservation. Currently, many native people in the Asia‐Pacific and Africa regions who maintain intergenerational connection to place and nature, Traditional Ecological Knowledge and manage resources similarly to Indigenous groups, sharing characteristics, for example, place‐based living, subsistence economies, but do not self‐identify as Indigenous or are not recognised as such legally or institutionally. Nonetheless, in achieving the 2030 goal of the Kunming–Montreal Global Biodiversity Framework for at least 30% of Earth's terrestrial, inland water and coastal and marine areas to be effectively conserved and managed, recognising and respecting the rights of IPLCs, including governance authority over traditional territories, would be crucial. Particularly in addition to an inclusive definition of IPLCs, the legal support for explicit recognition of Indigenous Peoples' land rights and fair and equitable participation would be key to the effective implementation of conservation outcomes. This is especially becoming more relevant within the context of global shifts towards nature‐based solutions, which require IPLCs perspectives in decision‐making and governance to conserve, restore and protect intact landscapes (Sangha, Ahammad, Russell‐Smith, Hernández‐Blanco, et al. 2025).

Finally, recognising Indigenous Peoples and their fundamental land rights contributes to maintaining place‐based cultural practices and knowledge required to monitor the culturally important species, conservation decision‐making and measures to enhance social‐ecological sustainability (Sterling et al. 2017). In some instances, conservation scientists and practitioners have increasingly aligned with Indigenous Peoples to co‐develop the knowledge for the identification, classification and protection of species by recognising Indigenous priorities of species that might otherwise be ignored in national policies on threatened species recovery (Goolmeer et al. 2022; Sangha, Ahammad, Russell‐Smith, and Wolley 2025). While Western science has generally focused on single species, Indigenous Peoples consider multi‐species for their various ecological functions and cultural relationship with them. So, co‐designing species protection requires the recognition of biocultural relationships (Lukawiecki et al. 2024). Indigenous Peoples' place, knowledge, practice and language are deeply interconnected, and recognising and defining these relationships can leverage the holistic conceptualisation of sustainability beyond conservation (Hill et al. 2020). An authentic engagement of Indigenous Peoples and recognising their relationships to land and integration of Indigenous knowledge and Western science will benefit conservation practices and outcomes while supporting just and culturally appropriate development opportunities for Indigenous Peoples.

## Author Contributions

**Ronju Ahammad:** conceptualization (equal), formal analysis (lead), methodology (equal), writing – original draft (lead), writing – review and editing (lead). **Kamaljit Sangha:** conceptualization (equal), funding acquisition (lead), supervision (lead), writing – original draft (equal), writing – review and editing (equal). **Jay Evans:** conceptualization (equal), writing – original draft (equal), writing – review and editing (equal). **Oscar Metcalfe:** conceptualization (equal), methodology (equal), writing – review and editing (equal).

## Funding Statement

Open access publishing facilitated by Charles Darwin University, as part of the Wiley – Charles Darwin University agreement via the Council of Australian University Librarians.

## Conflicts of Interest

The authors declare no conflicts of interest.

## Supporting information


**Data S1:** Indigenous Peoples' definitions and sources.

## Data Availability

All the required data are uploaded as Data S1. This paper presents authors' analysis based on their experience in the field and case studies; hence, most of the data is already cited and referred to in the paper.

## Endnotes

^1^
Tothi in Andhra Pradesh; Great Andamanese in Andaman and Nicobar Islands; Totos in West Bengal; Maram Naga in Manipur; Maria Gond in Maharashtra; and Hill Korbas and Abujh Maria in Madhya Pradesh.
