# Supplementary material for: Revisiting the Definition and Recognition of Indigenous Peoples and Local Communities for Biodiversity Conservation
Source: Ecol Evol. 2026 Feb 18;16(2):e72958. doi: 10.1002/ece3.72958 (PMC12917333; doi:10.1002/ece3.72958)
Supplement: Supplementary file 1 — Data S1: Indigenous Peoples' definitions and sources. [file ECE3-16-e72958-s001.docx]

**Supplementary materials**: Indigenous Peoples’ definitions and sources.

| Definitions | References |
| --- | --- |
| ILO provides an inclusive definition by considering the equal rights of Indigenous and Tribal Peoples.   - Tribal peoples in independent countries whose social, cultural and economic conditions distinguish them from other sections of the national community, and whose status is regulated wholly or partially by their own customs or traditions or by special laws or regulations; - Peoples in independent countries who are regarded as Indigenous on account of their descent from the populations which inhabited the country, or a geographical region to which the country belongs, at the time of conquest or colonisation or the establishment of present state boundaries and who, irrespective of their legal status, retain some or all of their own social, economic, cultural and political institutions. | Article 1 of ILO Convention 1989 (169) |
| UNDRIP is a significant international instrument that provides the principles for the protection of the rights of Indigenous Peoples with particular emphasis on self-determination.   - Indigenous Peoples have the right to self-determination. By virtue of that right they freely determine their political status and freely pursue their economic, social and cultural development. - Indigenous peoples have the right to maintain and strengthen their distinct political, legal, economic, social and cultural institutions, while retaining their right to participate fully, if they so choose, in the political, economic, social and cultural life of the State. | UNDRIP 2007, Article 3, Article 5 |
| Indigenous communities, peoples and nations are those which:   - Having a historical continuity with pre-invasion and pre-colonial societies that developed on their territories, consider themselves distinct from other sectors of the societies now prevailing in those territories, or parts of them. - They form at present non-dominant sectors of society and are determined to preserve, develop, and transmit to future generations their ancestral territories, and their ethnic identity, as the basis of their continued existence as peoples, in accordance with their own cultural patterns, social institutions and legal systems.   Historical continuity may consist of the continuation, for an extended period reaching into the present, of one or more of the following factors:  (a) Occupation of ancestral lands, or at least of part of them;  (b) Common ancestry with the original occupants of  these lands;  (c) Culture in general, or in specific manifestations  (such as religion, living under a tribal system, membership of an indigenous community, dress, means of livelihood, life-style, etc.);  (d) Language (whether used as the only language, as mother-tongue, as the habitual means of communication at home or in the family, or as the main, preferred, habitual, general or normal language);  (e) Residence in certain parts of the country, or in certain regions of the world;  (f) Other relevant factors. | Cobo 1987, Page 29 |
| Indigenous Peoples are the descendants of those who were there before others who now constitute the mainstream and dominant society. They are defined partly by descent, partly by the particular features that indicate their distinctiveness from those who arrived later, such as their language and ways of life, and partly by their own view of themselves.  A tribe is a distinct people, dependent on their land for their livelihood, largely self-sufficient, and not integrated into the national society. Although nearly all tribal peoples are also Indigenous, there are some who are not Indigenous to the areas where they live now. It’s important to make the distinction between tribal and Indigenous because tribal peoples have a special status acknowledged in international law as well as problems in addition to those faced by the wider category of Indigenous peoples.  Not all Indigenous Peoples are also tribal: the Quechua and Aymara of the Andes, for example, are the majority rural, agrarian population in Ecuador, Peru and Bolivia, and often integrated into the national economy. | Survival International 2024 |
| The term Indigenous Peoples is a common denominator for distinct peoples who, through historical processes, have been marginalised and denied their right to control their own development. For indigenous peoples, self-identification is the basic principle in claiming and asserting indigenous identity. Indigenous peoples present a vast spectrum of differentiated organisational representations depending on their history, relationship with the State, level of recognition and other contextual factors. | IWGIA 2018 |
| Indigenous Peoples and Local Communities (referred to by the acronym “IPLCs” in IPBES assessments) refer to  individuals and communities who either self-identify as indigenous or as members of distinct local communities, and that maintain an inter-generational historical connection to place and nature through livelihoods, cultural identity, languages, worldviews, institutions, and ecological knowledge. | IPBES 2022 |
